# 423. Mirror Mirror on the Wall Who is Driving all the Invasive GAS After All - The Utah Experience

**DOI:** 10.1093/ofid/ofae631.137

**Published:** 2025-01-29

**Authors:** Benjamin McMillion, Nicole L Pershing, Hillary Crandall, Adam Hersh, Krow Ampofo, Jared Olson, Kelly F Oakeson, Jennifer M Wagner, Shannon Nielsen, Mandy Dickey, Kelly Huynh, Andrew T Pavia, Anne J Blaschke

**Affiliations:** University of Utah School of Medicine, Salt Lake City, UT; University of Utah School of Medicine, Salt Lake City, UT; University of Utah School of Medicine, Salt Lake City, UT; University of Utah School of Medicine, Salt Lake City, UT; University of Utah, Salt Lake City, Utah; Primary Children's Hospital, Salt Lake City, Utah; Utah Department of Health & Human Services / Utah Public Health Laboratory, Taylorsville, Utah; Utah Department of Health and Human Services, Taylorsville, Utah; University of Utah School of Medicine, Salt Lake City, UT; Primary Children's Hospital, Salt Lake City, Utah; Intermountain Health, Salt Lake City, Utah; University of Utah, Salt Lake City, Utah; University of Utah School of Medicine, Salt Lake City, UT

## Abstract

**Background:**

*Streptococcus pyogenes* (GAS) is a common cause of non-invasive mucosal infections as well as rare life-threatening invasive disease. At the onset of the COVID-19 pandemic there was a decline in invasive GAS (iGAS) disease, followed by an increase in the fall of 2022 with the rise of *emm*12. Data on the association of circulating non-invasive and invasive *emm*-types are limited. We sought to characterize and compare *emm*-types of pharyngeal GAS to iGAS before and after the emergence of COVID-19.

**Figure 1:**
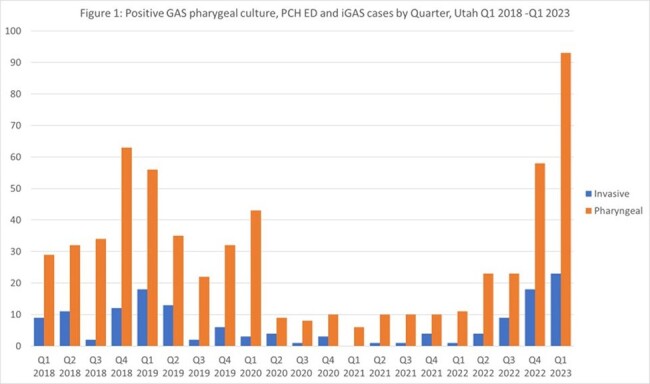
Positive GAS pharyngeal culture, PCH ED and iGAS cases by Quarter, Utah Q1 2018 - Q1 2023

**Methods:**

We retrospectively identified both pharyngeal and invasive GAS isolates obtained at Primary Children’s Hospital (Salt Lake City, Utah) from 2018-2023 using our GAS isolate database. Pharyngeal isolates were obtained primarily from our emergency department, while iGAS isolates were from hospitalized patients. Isolates underwent whole genome sequencing for *emm* typing, virulence factor identification, and phylogenetic analysis. We compared pharyngeal and invasive isolates for *emm*-type distribution over time and the presence of known virulence genes.

**Figure 2:**
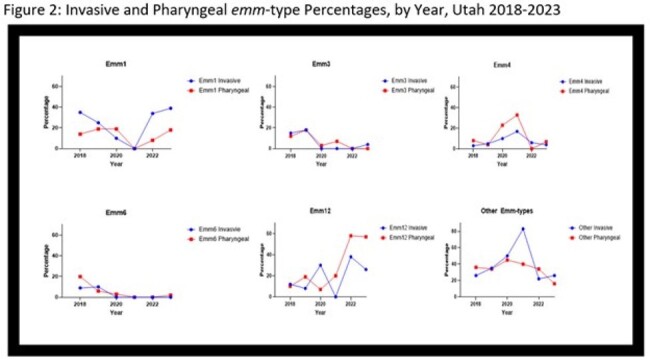
Invasive and Pharyngeal emm-type Percentages, by Year, Utah 2018-2023

**Results:**

From 1/2018 to 4/2023, we identified ∼650 pharyngeal GAS isolates, of which a random subsample of 306 were sequenced; 286 yielded sequences suitable for analysis. We identified 145 iGAS cases; all were sequenced. We observed a marked decrease in pharyngeal GAS from Q2 2020 through Q3 2022, followed by a steep increase which paralleled iGAS (Figure 1). The distribution of *emm*-types causing iGAS mirrored that of non-invasive pharyngeal isolates over time (Figure 2). Post-pandemic, *emm*12 and *emm*1 dominated both pharyngeal and invasive isolates. Comparing virulence genes between pharyngeal and iGAS isolates showed *sfbl/prtF1* were strongly associated with invasive disease in *emm*12 and *emm*59 (OR: 5.0, 95% CI [2.2-11]) while the presence of *sic* was strongly associated with invasive disease in *emm*1 (OR: 16.2, 95% CI [5.6-45.9]).

**Conclusion:**

After a marked decrease in GAS infections in children during the COVID-19 pandemic in Utah, there was a rebound of disease. Molecular analysis revealed invasive *emm*-types mirrored trends in pharyngeal isolates. Virulence genes *sfbl/prtF1* and *sic* were associated with invasive disease in *emm*12 and *emm1* respectively. Further analysis of evolutionary relatedness and virulence determinants is ongoing.

**Disclosures:**

**Krow Ampofo, MBChB**, Merck: Grant/Research Support **Andrew T. Pavia, MD**, GSK/Haleon: Advisor/Consultant|Sanofi: Advisor/Consultant **Anne J. Blaschke, MD, PhD**, BioFire Diagnostics/Biomerieux: I have IP owned by the U. of Utah licensed to BioFire and receive royalties.|Merck & Co: Advisor/Consultant

